# Sustained remission of rheumatoid arthritis with a specific serotonin reuptake inhibitor antidepressant: a case report and review of the literature

**DOI:** 10.1186/1752-1947-5-112

**Published:** 2011-03-19

**Authors:** Rajeev Krishnadas, Ranjit Krishnadas, Jonathan Cavanagh

**Affiliations:** 1 Sackler Institute of Psychobiological Research, University of Glasgow, Southern General Hospital, Glasgow, G51 4TF, UK; 2 Amrita Institute of Medical Sciences and Research Centre, Amrita Lane AIMS Ponekkara Post, Kochi, Kerala 682 041, India

## Abstract

**Introduction:**

The mainstay of pharmacologic therapy for rheumatoid arthritis includes the use of disease-modifying agents like sulfasalazine and methothrexate, and more recently, anti-tumor necrosis factor-α agents. Depression remains a major co-morbidity in patients with rheumatoid arthritis and is thought to contribute to disability and mortality in these patients. Evidence now suggests that a biologic link exists between substrates responsible for inflammatory conditions and mood disorders. Most of this evidence comes from preclinical studies. Nevertheless, more research into this area is helping us to understand the possible mechanisms through which these conditions interact with each other.

**Case presentation:**

We describe a 60-year-old Indian man with rheumatoid arthritis diagnosed 15 years ago who had minimal response to multiple therapies with disease-modifying agents and whose arthritis symptoms surprisingly remitted when he was started on a specific serotonin reuptake inhibitor antidepressant, three years ago, for co-morbid major depression. This remission has been maintained with this medication, and the patient is currently not taking any antirheumatoid medications.

**Conclusion:**

Possible mechanisms linking substrates of mood disorders and inflammation are reviewed in this case report, particularly the serotonergic system. Evidence seems to suggest a significant interaction between the serotonergic systems and inflammation. This interaction seems to be bidirectional. An understanding of this relation is most important to gain insight not only into pathophysiological mechanisms underlying this condition, but also into how treatments for these conditions may complement each other and possibly provide greater therapeutic options in both of these disabling conditions.

## Introduction

Rheumatoid arthritis (RA) is a chronic, disabling condition that primarily affects joints, producing an inflammatory synovitis that often progresses to destruction of the articular cartilage and ankylosis of the joints. Prevalence of RA is 1.16% in women and 0.44% in men in the United Kingdom [1]. A similar prevalence has been reported in India [2]. The mainstay of pharmacologic therapy for RA includes the use of disease-modifying agents (DMARDs) like sulfasalazine and methothrexate, and more recently, "biologic agents" like anti-TNF-α agents [3].

Conservative estimates suggest that major depressive disorder affects between 13% and 17% of patients with RA [4]. Major depressive disorder is thought to be an independent risk factor for both work disability and mortality in those with RA [5]. Clinical associations between medical illnesses and major depressive disorder are not solely attributable to illness-induced disability or pain. A growing body of evidence implicates mechanisms involved in a bi-directional link between biologic substrates of mood disorders and inflammation. Low-dose tricyclic antidepressants (TCAs) have long been a part of the armamentarium to treat pain and sleep disturbance in this population. Few studies have reported the effectiveness of specific serotonin reuptake inhibitors (SSRIs) in this condition.

Here we describe a patient with RA, whose disease remitted completely with an SSRI started for an episode of major depressive disorder.

## Case presentation

A 60-year-old Indian man (90 kg/170 cm) with a history of RA, presented with features suggestive of a severe depressive episode three years back. He had no previous or family history of depression. He was first diagnosed with RA fourteen years back and was treated with sulfasalazine and diclofenac for six years. He gradually developed resistance to these drugs, with persistent synovitis, progressing joint deformities, and elevated erythrocyte sedimentation rate (ESR) and C-reactive protein (CRP). An acute exacerbation of the illness occurred six years later, during which he was switched to a methotrexate regimen thrice weekly, with partial response. He developed severe anemia and related angina with methotrexate and had to discontinue methotrexate after three years. Since the episode of angina, the patient was started on a daily regimen of low-dose aspirin at 75 mg and simvastatin at 40 mg. He continued to take various non-steroidal anti-inflammatory drugs (NSAIDs) without much effect until three years back, when he presented to his clinician with an episode of severe depressive illness. He was referred to a psychiatrist, who started him on escitalopram, a serotonin-specific reuptake inhibitor, at 10 mg/day, and risperidone, an antipsychotic with a serotonin dopamine antagonist action at 1 mg/day. Risperidone was started, as the patient showed significant ruminative thoughts almost bordering on delusions. During this period, he was also taking aspirin and simvastatin at the previously mentioned doses.

Because the episode of depression was severe, it was decided to maintain him on the antidepressant medication for at least a year after complete remission of his depression. Before starting the patient on escitalopram and risperidone, his DAS 28 score was 6.6, suggesting that his arthritis was far from being under control. With the combination of escitalopram and risperidone, along with an improvement in his depressive symptoms, the patient also perceived significant improvement in his arthritis symptoms within a period of three to four weeks. Risperidone was gradually tapered and stopped after two months. His pain, morning stiffness, and fatigue continued to improve with escitalopram. Initially, the perception of improvement in arthritis symptoms was attributed to the improvement in his mood. However, his end-of-the-year rheumatology assessment showed that his DAS 28 scores had significantly improved to 2.2, being in the remission criteria range suggested for the Indian population [6]. After a period of one year taking escitalopram, it was decided gradually to taper and stop his antidepressants. On stopping the antidepressants, his joint pain and stiffness started to worsen, and in two weeks, at the patient's request, he was recommenced on 10 mg of escitalopram and has since been maintained on the same. His depressive illness and arthritis have been under remission since.

## Discussion

SSRIs have been found to be effective in treatment of depression in RA [7]. Recently Baune and Eyre [8] reported a case of RA that responded to a combination of SSRIs and antipsychotics. Our case differs from Baune and Eyre's report in that the patient was taken off the risperidone (antipsychotic) in two months, but continued to maintain his remission. More interesting is that the patient continues to be under remission despite not taking any disease-modifying agent or anti-inflammatory medications. The patient was taking the combination of aspirin, 75 mg, and simvastatin, 40 mg, for almost five years before he was commenced on the psychotropic medications. No perceived improvement in arthritis was noted during this period. A clear temporal association was found between the start of escitalopram and the improvement in his arthritis. Further, his arthritis relapsed when his escitalopram was stopped, and improved when it was restarted. Therefore, the improvement in the patient's inflammatory condition could be attributed to the psychotropic medication rather than to the aspirin or the simvastatin.

A bi-directional relation seems to exist between biologic substrates of mood disorders and inflammation. For example, inflammatory mediators like proinflammatory cytokines are thought to have a direct impact on biologic substrates implicated in the pathophysiology of mood, particularly the serotonergic system, and conversely, serotonergic pathways are thought to be important in mediating both inflammation and mood.

### Inflammation modulates serotonergic system

#### Inflammation upregulates serotonin transporter

A key site of action of antidepressants is the serotonin transporter (SERT), which regulates serotonergic neurotransmission. Data from preclinical studies indicate that both the density and the activity of SERT are increased by proinflammatory cytokines (for example, TNF-α), leading to an increase in 5-HT uptake from the synapse, thus decreasing 5-HT transmission [9]. This regulation of neuronal SERT activity occurs via p38 mitogen-activated protein kinase-linked pathways. Data from our group confirms this hypothesis in humans. Treatment with TNF blockade agent adalimumab led to a decrease in serotonin-transporter binding by up to 20% by using [^123^I]β-CIT-SPECT in a group of patients with rheumatoid arthritis [10].

#### Inflammation activates the kynurenine pathway

Some evidence suggests that proinflammatory cytokines, including TNF-α, induce glial indoleamine 2,3-dioxygenase (IDO). This activates the kynurenine pathway, thus channeling the available tryptophan to form kynurenine (Kyn), 3-hydroxykynurenine (3HK), and quinolinic acid (QUIN), rather than the serotonin (5-HT). This leads to a decrease in the availability of 5-HT, thus contributing to the serotonin-depletion hypothesis of depression. Further, 3HK and QUIN are NMDA-receptor agonists. High concentrations of these compounds lead to excitotoxicity and calcium-mediated cell death. Taken together, some data support the hypothesis that IDO pathway modulation plays a role in the pathophysiology of cytokine-induced depression [11].

#### Anti-inflammatory agents have antidepressant properties

Muller *et al*. [12] showed that addition of celecoxib, a COX-2 inhibitor that inhibits prostaglandin E2 to reboxetine (a norepinephrine-reuptake inhibitor), showed significant additional effects on depressive symptoms compared with reboxetine alone. Specific TNF-blockade agents have been shown to improve mood, independent of improvement in the inflammatory condition. Tyring *et al. *[13] found that 55% of patients with psoriasis who were treated with etanercept showed a 50% reduction in their Beck Depression Inventory (BDI) scores compared with 39% taking placebo, an effect size comparable to that of antidepressants. Analyses of the individual items of the BDI showed that significant improvements at week 12 were seen in feelings of guilt, irritability, anhedonia, sleep, and sexual symptoms. All of them are deemed to be core depressive symptoms. They also found that improvement in depression scores did not correlate with improvement in joint pain, skin pain, or itching, suggesting that the improvement in mood was independent of improvement in psoriasis.

### Serotonergic systems modulate inflammation

#### Descending serotonergic pathways modulate inflammatory pain

Descending spinal serotonergic pathways from the medulla have long been implicated in the physiology of pain modulation. Zhao *et al*. [14] showed that knockout mice that lacked these descending serotonin pathways in the brain showed normal thermal and visceral pain responses but were less sensitive to mechanical stimuli and exhibited enhanced inflammatory pain compared with their littermate control mice. More specifically, they showed that the analgesic effects of antidepressants were absent in this strain of mice, suggesting that serotonergic pathways play an important role in modulating inflammatory pain, compared with mechanistic pain.

#### Antidepressants have anti-inflammatory and analgesic properties

Antidepressants with a dual action (inhibiting serotonin and norepinephrine reuptake) have been shown to have analgesic properties and are recommended first-line treatments in a number of painful conditions [15]. Tricyclic antidepressants in low doses have been used regularly in rheumatology clinics for their effect on pain, mood, and sedation. Antidepressants also were shown to induce an anti-inflammatory response, independent of the antidepressant action. O'Brien *et al*. [16] showed that C-reactive protein (CRP) levels decreased after treatment with an antidepressant. This effect was independent of its antidepressant effect. Vollmar *et al. *[17] found that venlafaxine significantly decreased clinical symptoms of disease in a murine autoimmune encephalomyelitis model. They showed that venlafaxine suppressed the generation of pro-inflammatory cytokines IL-12 p40, TNF-α, and IFN-γ in encephalitogenic T-cell clones, splenocytes, and peritoneal macrophages *in vitro*. Piletz *et al. *[18] found that increased pro-inflammatory biomarkers in patients with major depressive disorder showed a decrease in response to treatment with venlafaxine (a mixed serotonin and norepinephrine-reuptake inhibitor, exhibiting serotonin-reuptake inhibition at lower doses, and norepinephrine-reuptake inhibition at higher doses) at the serotonergic dose range rather than at the norepinephrine dose range, suggesting that sertonergic pathways mediate the anti-inflammatory response to anti-depressants. More recently, Sacre *et al. *[19] found that fluoxetine and citalopram significantly inhibited disease progression in murine collagen-induced arthritis (CIA) models. Both drugs were also found to inhibit significantly the spontaneous production of tumor necrosis factor, IL-6, and IFN-γ-inducible protein 10 in human RA synovial membrane cultures. The potential mechanism through which fluoxetine and citalopram treatment exhibited these anti-inflammatory effects was explored. Both the drugs significantly inhibited the signaling of Toll-like receptors 3, 7, 8, and 9, providing a potential mechanism for their anti-inflammatory action. Toll-like receptors are proteins thought to mediate innate immunity. They play an important role in initiating an inflammatory reaction in response to pathogen proteins and endogenous molecules found at sites of inflammation and tissue damage.

#### 5-HT2A receptors mediate the inflammatory response to serotonin

Recent animal and human data suggest that certain subtypes of serotonin receptors may play a role in mediating inflammatory processes. 5-HT2A receptors are expressed widely throughout the central nervous system. In the periphery, they are highly expressed in platelets and many cell types of the cardiovascular system, in fibroblasts, and in neurons of the peripheral nervous system. Yu and colleagues [20] found that peripheral activation of 5-HT2A receptors in primary aortic smooth muscle cells leads to an extremely potent inhibition of tumor necrosis factor (TNF)-α-mediated inflammation, a possible mechanism of action of SSRIs in mediating the anti-inflammatory action. Interestingly, they found that proinflammatory markers could also be inhibited by 5-HT2A stimulation hours after treatment with TNF-α (that is, after the onset of inflammation).

SSRIs, including escitalopram, are thought to increase extracellular serotonin concentrations at these receptors. However, SSRIs are thought to downregulate 5-HT2A in the long run. Surprisingly, blockade of 5-HT2A receptors (risperidone) also has the same effect (that is, downregulation). This may seem paradoxical. Meyer *et al. *[21] suggested that treatment with SSRIs led to a decrease in 5-HT2A binding potential, suggesting a decrease in receptor density over a period of six weeks. They found that this decrease in binding potential became less pronounced with increasing age, suggesting that downregulation of 5-HT2A receptors decreased with age. This observation was thought to be due to a possible floor effect caused because the 5-HT2A-receptor density decreased with age. Nevertheless, the 5-HT2A receptor downregulation suggests that SSRIs do have an effect on 5-HT2A receptors. The fact that this downregulation was less in older individuals in Meyer's study means that the 5-HT2A stimulation would continue without significant downregulation, possibly leading to a powerful anti-inflammatory effect peripherally in these individuals, a possible reason that escitalopram had this effect in the individual in the report. Whether this downregulation is essential for the anti-inflammatory effect must be investigated further.

In addition, it has been postulated that people taking antidepressants that blocked 5-HT2A receptors are 45 times more likely to report an adverse drug reaction pertaining to a joint, compared with those that did not block these receptors, confirming the hypothesis that 5-HT2A receptors play an important role in mediating inflammatory processes [22].

## Conclusion

In the present case, we see that treatment of co-morbid depression with an SSRI led to complete remission of arthritis in a 60-year-old individual. Postulated mechanisms through which antidepressants mediate this effect include their agonistic action on 5-HT2A receptors or inhibition of the signaling of Toll-like receptors that are responsible for mediating innate immunity. The relation between mediators of inflammation and biologic substrates of mood seem to be bidirectional. Further studies are required to elucidate the mechanisms involved in this relation.

## Abbreviations

3HK: 3-hydroxy kynurenine; 5-HT: serotonin; 5-HT2A: 5-HT2A subtype of serotonin receptor; BDI: Beck Depression Inventory; CIA: collagen-induced arthritis; CRP: C-reactive protein; DAS 28: disease activity score-28; DMARDs: disease-modifying anti-rheumatic drugs; ESR: erythrocyte sedimentation rate; IDO: indoleamine 2,3-dioxygenase; IFN-γ: interferon-γ; IL-12: interleukin 12; Kyn: kynurenine; NMDA: *N*-methyl-d-aspartate; NSAID: non-steroidal anti-inflammatory drug; QUIN: quinolinic acid; RA: rheumatoid arthritis; SERT: serotonin transporter; SPECT: single-photon emission computed tomography; SSRI: specific serotonin-reuptake inhibitor; TCA: tricyclic anti-depressant; TNF-α: tumor-necrosis factor-α.

## Competing interests

The authors declare that they have no competing interests.

## Authors' contributions

RVK was involved in collating the information, review of literature, and preparation of the manuscript. RTK was involved in collating information regarding the case and getting informed consent from the patient. JC was involved in review of literature and revising the manuscript critically. All authors read and approved the final manuscript.

## Consent

Written informed consent was obtained from the patient for publication of this case report and accompanying images. A copy of the written consent is available for review by the Editor-in-Chief of this journal
